# A Novel Biomarker Identification Approach for Gastric Cancer Using Gene Expression and DNA Methylation Dataset

**DOI:** 10.3389/fgene.2021.644378

**Published:** 2021-03-25

**Authors:** Ge Zhang, Zijing Xue, Chaokun Yan, Jianlin Wang, Huimin Luo

**Affiliations:** School of Computer and Information Engineering, Henan University, Kaifeng, China

**Keywords:** gastric cancer, omics data, biomarkers, feature selection, deep neural network, machine learning

## Abstract

As one type of complex disease, gastric cancer has high mortality rate, and there are few effective treatments for patients in advanced stage. With the development of biological technology, a large amount of multiple-omics data of gastric cancer are generated, which enables computational method to discover potential biomarkers of gastric cancer. That will be very important to detect gastric cancer at earlier stages and thus assist in providing timely treatment. However, most of biological data have the characteristics of high dimension and low sample size. It is hard to process directly without feature selection. Besides, only using some omic data, such as gene expression data, provides limited evidence to investigate gastric cancer associated biomarkers. In this research, gene expression data and DNA methylation data are integrated to analyze gastric cancer, and a feature selection approach is proposed to identify the possible biomarkers of gastric cancer. After the original data are pre-processed, the mutual information (MI) is applied to select some top genes. Then, fold change (FC) and *T*-test are adopted to identify differentially expressed genes (DEG). In particular, false discover rate (FDR) is introduced to revise *p*_value to further screen genes. For chosen genes, a deep neural network (DNN) model is utilized as the classifier to measure the quality of classification. The experimental results show that the approach can achieve superior performance in terms of accuracy and other metrics. Biological analysis for chosen genes further validates the effectiveness of the approach.

## 1. Introduction

Gastric cancer is one of the most common malignant tumors of the digestive system (Nogueira et al., 2017). The pathogenesis is mainly relevant to helicobacter pylori infection, diet, environment, and genetic factors. It remains one of the most deadly cancers worldwide, especially among older males (Siegel et al., 2020). Generally speaking, early detection of cancer is crucial for increasing the chances for successful treatment and prolonging the patient's life. The 5-year survival rate of early-stage gastric cancer can reach more than 95% (Song et al., 2017). However, the early stage of gastric cancer is hard to monitor because of rare symptoms and some potential patients' cancer may be advanced when they are first diagnosed. Therefore, early targeting and treatment are very important in clinical practice of gastric cancer (Wang et al., 2020). In recent years, with the development of sequencing technology, the genome data of cancer patients can be obtained easily. These genomic data have been used to study the association between genetic changes and diseases and contribute to diagnosis and prognosis. However, these data always have the characteristics of high dimensions and low sample size (HDLSS) (Han et al., 2019). It is hard to process these data directly (Yan et al., 2018). Therefore, feature selection technology is usually adopted to assist in analyzing the possible cancer-causing genes, also called biomarkers, from massive cancer data. The biomarkers can facilitate us to understand the pathogenesis of diseases at a detailed molecular level and play an auxiliary role in clinical diagnosis.

Till now, many researchers have applied the feature selection methods to the field of gene expression data analysis (Ding and Peng, 2005; Lu et al., 2017; Zhao et al., 2020). However, it is incomprehensive to analyze cancer only using gene expression data. The rapid accumulation of omics data can provide disparate, partially independent, and complementary information about the entire genome (Zhang et al., 2016). The multi-omic data can lay an important foundation for mining informative biomarkers for cancer (Ruffalo et al., 2015). Among these omics data, DNA methylation is an important epigenetic event that affects gene expression during the development in various diseases such as cancer (Bird, 1986; Wang et al., 2018). In general, DNA methylation status is more reliable than gene expression (Paziewska et al., 2014). The combination of DNA methylation data and gene expression data is more beneficial to explain the pathogenesis of gastric cancer. Therefore, these two kinds of data are utilized to identify the biomarkers of gastric cancer in our study.

In this paper, we propose a novel gastric cancer biomarker identification approach, referred to GCBMI, to discover the possible biomarkers of gastric cancer. First, the gene expression data and DNA methylation data of gastric cancer are collected and processed. Then, fold change, statistical test, and mutual information are utilized to identify the differentially expressed genes of gastric cancer and the selected genes can serve as guidelines to reduce the dimension of omics data. At last, the DNN model is adopted as the classifier to measure the quality of classification. Experimental results indicate that GCBMI can obtain more favorable performance than other state-of-art methods.

The main contributions of this study are summarized as follows:

- For gastric cancer, a novel feature selection approach is proposed to identify the potential biomarkers. Here, DNA methylation data is integrated with the gene expression data effectively to obtain a comprehensive analysis to discover the relationship between gastric cancer and potential biomarkers.
- Besides *T*-test and FC, mutual information is introduced as a preliminary screening method to filter out redundant genes and FDR is adopted to revise *p*_value to further screen genes.
- The experimental results suggest that our approach can achieve improvement in different evaluation indicators than other state-of-art methods. In addition to evaluating accuracy, GO analysis, heatmap, and literature review are executed. The above biological validation is able to demonstrate that the genes selected by our approach are associated with gastric cancer.

The remainder of this paper is organized as follows: In section 2, we review related works of feature selection methods. The proposed approach is introduced in section 3. section 4 introduces the experimental design. Experimental results and biological analysis are described in section 5. Finally, we summarize the paper and make a vision for the future in section 6.

## 2. Related Work

With the development of sequencing technology, massive amounts of cancer genome data have been accumulated at an accelerated speed. A number of feature selection methods have been extensively applied to cancer data. Traditional feature selection methods can be divided into two categories: filter methods and wrapper methods. Among them, the filter method has the advantage of low time consumption. So far, some filter methods had been well-applied to gene expression data.

Principal Component Analysis (PCA) is an effective dimensionality reduction method (Wold et al., 1987). Ding et al. combined feature extraction with feature selection in gene expression data (Ding et al., 2009). The relief was utilized to feature selection, and PCA was used to extract features. Then, they used the support vector machines (SVM) for classification. Experimental results illustrated that their method is effective to reduce the classification error rate in eight cancer datasets. But such methods cannot guarantee that the features still remain the corresponding biological significance. For example, the dimensionality reduction of features by PCA is equivalent to mapping the new features on the original features, and the features obtained after PCA are different from the original genes (Shen and Huang, 2008). Thus, it is often difficult to interpret the results.

Hsu et al. used extremely randomized trees (ET) to calculate the weight of the features (Hsu and Si, 2018). Feature selection was achieved by selecting features with high weight. Then, the linear SVM was combined to achieve about 95% accuracy on TCGA datasets. Lee et al. developed a novel filter method to identify the biomarkers of lung cancer and confirmed seven possible biomarkers (Lee et al., 2011).

In addition to filter methods, the wrapper methods utilize classification accuracy as a measurement standard for evaluation and find the optimal feature subset by iteration of meta-heuristic algorithms (Rodrigues et al., 2014). A lot of meta-heuristic algorithms had been well-applied to wrapper methods for feature selection of cancer such as bat algorithm (BA), recursive memetic algorithm (RMA), binary krill herd algorithm (MBKH), and so on (Dashtban et al., 2018; Ghosh et al., 2019; Zhang et al., 2020).

Dashtban et al. proposed MOBBA-LS which utilized fisher criterion and BA (Dashtban et al., 2018). They tested their method on three microarray cancer datasets. The accuracy achieved 100, 97, and 100% on leukemia, prostate, and SRBCT datasets, respectively. Ghosh et al. developed a recursive memetic algorithm (RMA) model for feature selection (Ghosh et al., 2019), and Zhang et al. proposed a pre-screening method of feature ranking, IG-MBKH, which is based on information gain (IG) and an improved binary krill herd (MBKH) (Zhang et al., 2020). The above methods can obtain favorable classification accuracy on microarray data of cancer.

Multiple-omics data can enable to provide a more comprehensive analysis of the entire genome. Among them, DNA methylation is one of the important epigenetic regulatory mechanisms (Luo et al., 2020). Especially, it is considered as a molecular factor that controls and regulates gene expression levels near the CpG sites. Its status is closely associated with diverse diseases and is generally more stable than gene expression (Ding et al., 2019). Therefore, the function of DNA methylation data was widely recognized. Increasing feature selection methods, which are based on gene expression data and DNA methylation data, were proposed.

For Alzheimer's disease, Park et al. proposed a biomarker prediction model, which integrated multi-omic data (Park et al., 2020). They used the Limma package to select possible biomarkers. Experimental results showed that their method can achieve better accuracy than using single data, and some chosen genes were reported in AlzGene database.

Mallik et al. proposed a method to identify biomarkers of cancer based on omics data (Mallik et al., 2017). The maximal relevance and minimal redundancy (mRMR) and parameter test like *T*-test were used to select the genes. The results suggested that their method had stable performance on different classifiers and classification accuracy can achieve about 95 and 90% in gene expression data and DNA methylation data, respectively.

Wang et al. proposed a feature selection method based on gene expression data and DNA methylation data of the six types of cancer (Wang et al., 2020). Their method can be divided into three steps. First, the correlation between gene expression profile and methylation profile of each gene was calculated to screen genes initially. Then, the genes were further filtered by *T*-test and FDR value. Finally, the genes selected in first two steps are filtered by Elastic Net. Finally, support vector machine was utilized as the classifier. The accuracy can be as high as 98% for the training set and 97% for the independent test set.

## 3. The Proposed Approach

**Figure 1:**
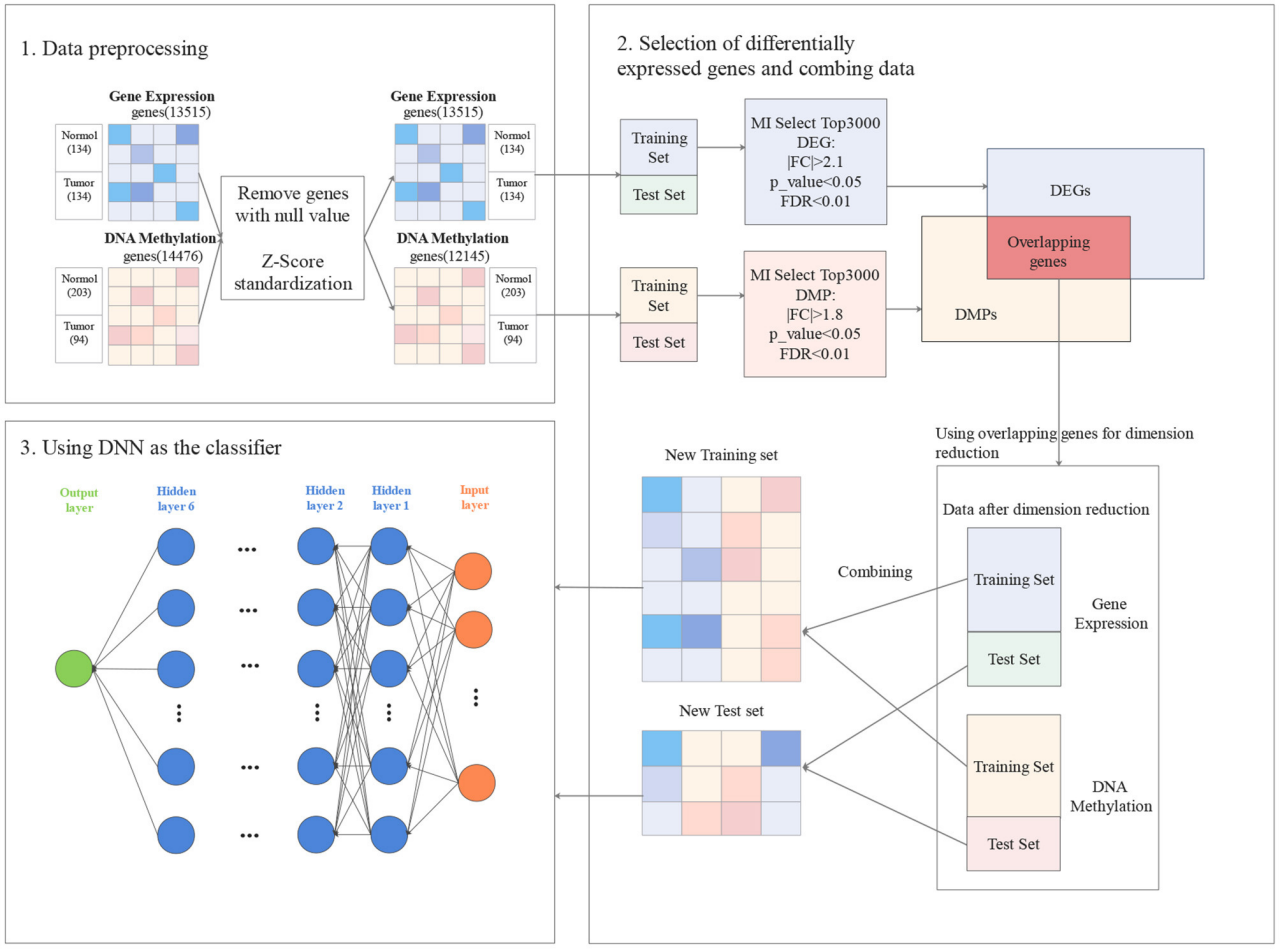
The workflow of gastric cancer biomarker identification approach (GCBMI).

In this section, the proposed approach GCBMI is introduced. The overall workflow of GCBMI is shown in Figure 1. GCBMI consists of three stages: data pre-processing, selection of DEG and data combination, and using deep neural network as the classifier.

### 3.1. Data Pre-processing

In this section, we regularize the gene expression data, and then merge the individual gene expression data files. In addition, on the basis of annotation file of the gene chip, the column (feature) name of each sample is converted to the gene name, and the label column is added. In the annotation file of the gene chip, the gene name corresponding to each probe is stored. If a gene corresponds to multiple probes, we take the median of expression value as new expression value of the gene. After that, the genes with null values are further removed. In order to eliminate the influence of outliers, the dataset is standardized by z-score according to the following formula (Zhang et al., 2014). Finally, the datasets are divided into training set and test set in our experiment.

(1) $$\begin{array}{l} x^{\prime }=\frac{x-\bar{x}}{\sigma } \end{array}$$

where x and *x*′ represent a column of data before and after standardization. $\bar{x}$ and σ represent the mean and standard deviation of a column of data in training set.

Likewise, DNA methylation data are also processed accordingly to eliminate the influence of outliers.

### 3.2. Selection of Differentially Expressed Genes and Data Combination

In this section, how to identify DEG in our approach is introduced. For gene expression data, the characteristics of high dimension and low sample size make it hard to construct a prediction model directly and may lead to the over-fitting (Ma and Zhang, 2019). For this issue, an appropriate method is required to reduce the size of feature space and the risk of over-fitting.

In GCBMI, the DEG and the differentially methylated positions (DMP) are utilized to train the model. The overall process contains three steps as follows.

First, MI (Liu H. et al., 2009) is applied to select TopN genes for gene expression data and DNA methylation data, respectively. It is a classic filter method of feature selection, which has been successfully applied to many feature selection problems (Peng and Fan, 2017). In order to avoid redundancy, the MI is adopted to filter out irrelevant genes. *N* is set to 3,000 through the subsequent experiments.

Second, FC and *T*-test are adopted to do identify DEG and DMP. What is more, the FDR is applied to revise the *p*_value. Taking DEG as an example, FC value for each selected genes in the first step is calculated. Since the data obey the normally distributed by Z-score standardization. Parametric statistics like *T*-test can work well on this kind of data. Then, Levene-test (Ankarali et al., 2009) is applied to verify whether the samples with variance homogeneity or not. If they have variance homogeneity, performing the standard *T*-test (Gauvreau and Pagano, 1993) to calculate *p*_value. Otherwise, the Welch's *T*-test (Algina et al., 1994) is executed to calculate the *p*_value. After that, the FC value and significant *p*_value for each gene are obtained. Finally, FDR is utilized to revise *p*_value to further screen candidate genes. A suitable threshold for FC value, *p*_value, and FDR are set to filter genes. And then we can obtain DEG. Similarly, DMP can be obtained. As shown in Figure 1, in gene expression data, the |*FC*| > 2.1 and *p*_value < 0.05.The |*FC*| > 1.8 and *p* < 0.05 in DNA methylation data. The FDR threshold value of both experimental datasets is set as 0.01. A hypothesis is made that if the gene is differentially expressed and occur hypermethylated and hypomethylated in different samples. This gene may have a potential relationship with gastric cancer. So the overlapping genes in DEG and DMP are the possible biomarkers of gastric cancer.

**Figure 2:**
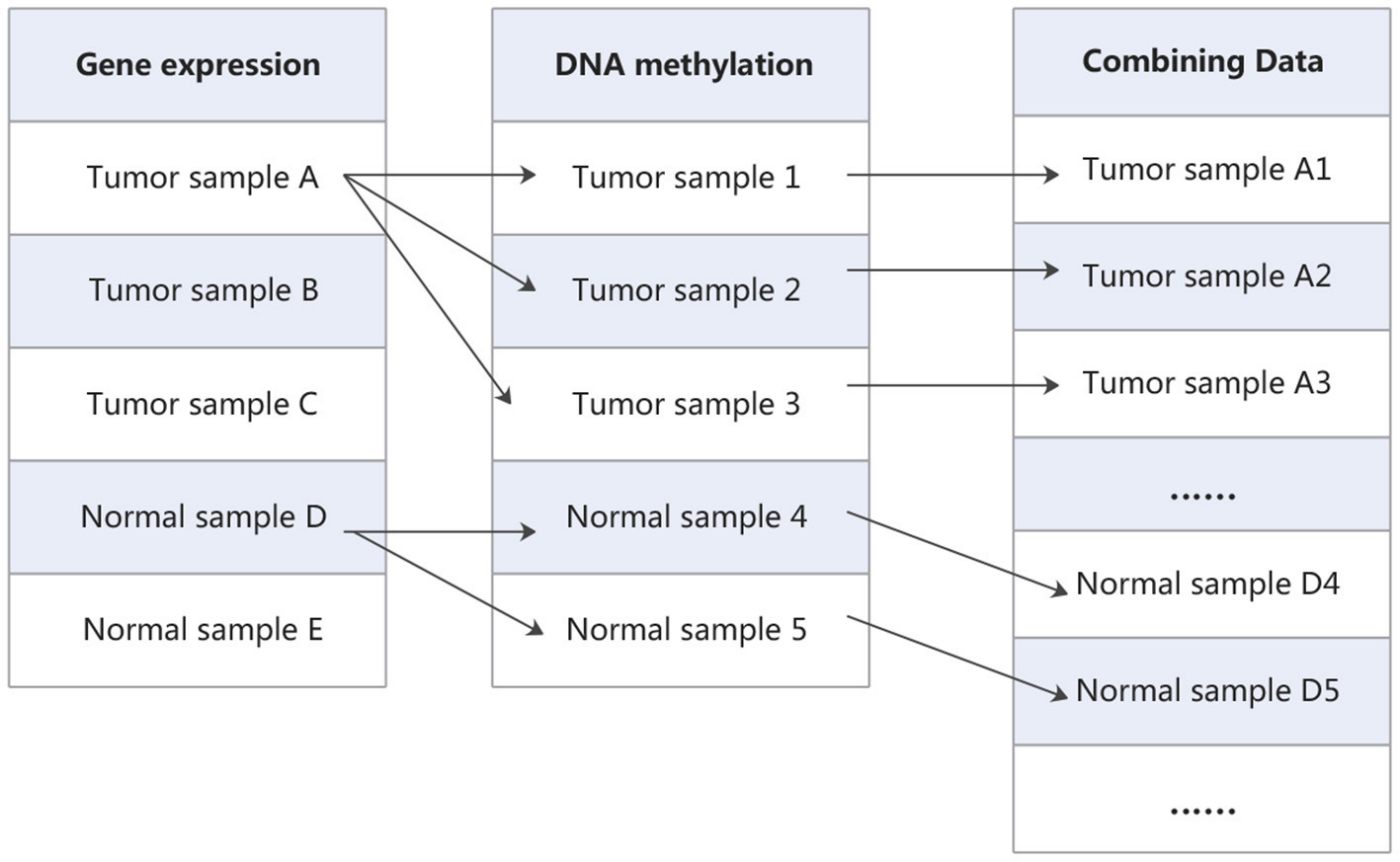
The process of combining data.

Finally, in order to extend training samples, all possible pairs of gene expression data and DNA methylation data for tumor and normal samples are utilized to merge into a new dataset. As shown in Figure 2, Cartesian product (Emelyanov and Ponomaryov, 2017) is performed on the gene expression data and DNA methylation data. The gene expression data and methylation data that labeled as tumor are combined into new tumor samples, and which labeled as normal are combined into new normal samples. In this way, the gene expression matrix and DNA methylation matrix are combined into a new expression matrix. This matrix has a large sample size. For example, in one of the cross-validation, the training set of gene expression data has 214 samples, which contains 112 tumor samples and 102 normal samples. DNA methylation data have 237 samples, which contains 160 tumor samples and 77 normal samples. After the combination, we will obtain 17,920 tumor samples and 7,854 normal samples. Taking them as new tumor samples and normal samples, so the new training set contains 25,774 samples, including 17,920 tumor samples and 7,854 normal samples.

### 3.3. Using Deep Neural Network as the Classifier

DNN model has excellent classification performance compared with traditional classifiers in previous studies, such as (Chen et al., 2020; Singh and Yamada, 2020). Here, the DNN also adopted as the classifier and the parameters of the DNN are determined through experiments.

In this section, the structure of the network is introduced. Our DNN model consists of three parts: input layer, hidden layer, and output layer. The input layer consists of two parts, corresponding to gene expression data and DNA methylation data, respectively. Then we add six hidden layers that applied ReLU as the activation function. Each layer contains 100 nodes and a additional bias nodes. The dropout is added for each hidden layer to avoid overfitting, which refers to drop some neurons randomly according to a certain probability during the learning iteration. It is equivalent to train a sparser network than the original network. Each of iterations is training a different network model to prevent overfitting. Finally, since our data only have two categories, the output layer with one node is sufficient. Sigmoid function is adopted as the activation function of the output layer to make the output value between 0 and 1.

In the DNN model, the loss function is binary cross entropy and cost function is the reduced average value of cross entropy. Adam algorithm is applied to optimize the parameters of the network model. The formula of the loss function and cost function are as follows:

(2) $$\begin{array}{l} L\left(ŷ,y\right)=-ylog\left(ŷ\right)-\left(1-y\right)log\left(1-ŷ\right) \end{array}$$

(3) $$\begin{array}{l} J(w,b)=\frac{1}{m}\sum_{i=1}^{m}(-y^{i}log({\hat{y}}^{i})-(1-y^{i})log(1-{\hat{y}}^{i}) \end{array}$$

where *y* and ŷ represent the true value and the predicted value of a sample. ŷ is the result of sigmoid regression. *m* is the total number of samples and *i* represents the index of the sample. *w* and *b* represent weights and biases, respectively.

## 4. Experimental Setting

The experiments can be divided into two parts. First, we compare GCBMI with other state-of-art methods. The ET (Hsu and Si, 2018), Elastic Net (Wang et al., 2020), IG-MBKH (Zhang et al., 2020), and MOBAA-LS (Dashtban et al., 2018) are selected as the baselines. A detailed description of the comparison methods is as follows:

- ET was proposed by Hsu et al. They used ET to calculate the weight of the features and select features with high weight. SVM was combined to evaluate the feature subsets. This method achieved about 95% accuracy on TCGA datasets.
- Elastic Net was a novel method that integrates the Pearson correlation coefficient, *T*-test, and FDR. The data are based on gene expression data and DNA methylation data. In six types of omics-data, the accuracy can up to about 98% by combing with SVM.
- IG-MBKH was presented and applied to feature selection for high-dimensional datasets. This method combined IG and krill herd algorithm and they used K-Nearest Neighbor (KNN) classifier to evaluate the classification accuracy. The accuracy of classification on nine different cancer datasets was more than 90%.
- MOBAA-LS is based on fisher criterion and BA. The accuracy achieved 100, 97, and 100% on leukemia, prostate, and SRBCT datasets, respectively.

Second, we investigate the prediction performance of DNN in biomarker identification for gastric cancer and how our method using different classifiers can affect the classification accuracy. We undertake experiments to compare our method using DNN classifier compared with using the traditional classifiers, such as KNN (Tahir et al., 2007), SVM (Vieira et al., 2013), and Naive Bayesian (NB) (Bielza and Larrañaga, 2014).

### 4.1. Dataset

We select the GEO database, which is an authoritative database of cancer applied in many previous studies (Zouridis et al., 2012; Wang et al., 2013) as the benchmark database. And the gene expression data GSE29272 (Li et al., 2014) and DNA methylation data GSE30601 (Lei et al., 2013; Kurashige et al., 2016) of gastric cancer are downloaded to construct our experiment dataset. As shown in Table 1, there are 268 samples of gene expression data including 134 tumor samples, 134 normal samples, and 13,515 features. And DNA methylation data contains 203 tumor samples, 94 normal samples, and 14,476 features.

**Table 1:** Benchmark dataset.

**Dataset**	**Gene expression**	**DNA methylation**
GEO ID	GSE29272	GSE30601
Normal samples	134	203
Tumor samples	134	94
Features	13515	14476

### 4.2. Parameter Setting

The experiments are conducted on Intel Dual Core CPU, 8 GB RAM, Windows 7 operating system. The procedure is implemented under the programming environment Python version 3.6. The feature selection algorithms, statistical detection methods, and classifiers are provided by the Scikit-learn package and scipy package and the DNN is built by Keras package. Related parameters are given as follows: DNN is set as described in the Section 3.3; SVM: degree = 3, gamma = auto, kernel = “rbf,” cache_size = 200; KNN: *K* = 5. The parameters of methods are set according to the original literature (Dashtban et al., 2018; Hsu and Si, 2018; Wang et al., 2020; Zhang et al., 2020). The specific settings are shown in Table 2.

**Table 2:** Parameter setting.

**Methods**	**Parameter setting**
GCBMI	MI: *n* = 3,000; Gene expression: |*FC*| > 2, *p* < 0.05, FDR < 0.01; DNA methylation: |*FC*| > 1.8, *p* < 0.05, FDR < 0.01
ET	Default parameters
IG-MBKH	*N* = 20; Iterations = 400; TopM = 80; Nmax = 4; Vf = 0.02; Dmax = 0.005
Elastic Net	*p* < 0.05, FDR < 0.01, ElasticNetCV (cv = 10)
MOBBA-LS	opN = 500, Population = 20, iteration = 300, alpha = 0.9, sigma = 0.7, injRate = 0.01, extRate = 0.01

According to Park et al. (2020), all experiments use five-fold cross validation. The dataset is divided into five parts, and one part is taken as the test set in order and the rest parts are taken as the training set in each cross validation. After the Cartesian product is executed, there are average 8,053 normal samples, 17,400 tumor samples as training set, and 496 normal samples, 1,079 tumor samples as test set. The accuracy, precision, recall, F1-score and Area Under Curve (AUC) are utilized to evaluate the classification results of the model (Tanzi et al., 2020). These evaluation indicators are defined as follows:

(4) $$\begin{array}{l} Accuracy=\frac{TP+TN}{TP+FP+TN+FN} \end{array}$$

(5) $$\begin{array}{l} Prediction=\frac{TP}{TP+FP} \end{array}$$

(6) $$\begin{array}{l} Recall=\frac{TP}{TP+FN} \end{array}$$

(7) $$\begin{array}{l} F1-Score=\frac{2\cdot Prediction\cdot Recall}{Prediction+Recall} \end{array}$$

The positive samples are tumor samples and the negative samples are normal samples. True positive (TP) indicates the number of tumor samples that have been correctly classified, false positive (FP) indicates the number of normal samples which are misclassified as tumor samples, true negative (TN) indicates the number of correctly classified normal samples, and false negative (FN) indicates the number of tumor samples, which are misclassified as normal samples.

## 5. Results and Discussion

### 5.1. Comparison of Other State-of-Art Methods

In this section, GCBMI is compared with other state-of-art methods, and the experimental results are shown in Table 3. The accuracy of GCMBI achieved is 98.7%. The Elastic net also applies omics data, but the accuracy of GCBMI is 9% higher than the Elastic net. The performance of two wrapper methods IG-MBKH and MOBBA-LS are similar in our experiment. In terms of accuracy, these two methods are about 5% lower than our approach. The accuracy of extremely randomized trees achieved is 93%. What is more, in terms of precision and recall, GCBMI also has the highest precision and the second highest recall. This indicates FP and FN appear less frequently and the classification performance of GCBMI is superior to other state-of-art methods.

**Table 3:** Performance comparison on different metrics (the accuracy, precision, recall, F1-score, and AUC value are average).

**Methods**	**Accuracy**	**Precision**	**Recall**	**F1-score**	**AUC**
GCBMI + DNN	**0.9870**	**0.9971**	0.9836	**0.9903**	**0.9891**
ET + SVM	0.9259	0.8571	**1.0**	0.9230	0.9333
Elastic Net + SVM	0.8922	0.9003	0.9433	0.9210	0.8598
IG-MBKH + KNN	0.9518	0.9730	0.9166	0.9437	0.9483
MOBBA-LS + SVM	0.94	0.9477	0.9327	0.9401	0.9412

*The bold values represent the highest value of each metrics*.

**Figure 3:**
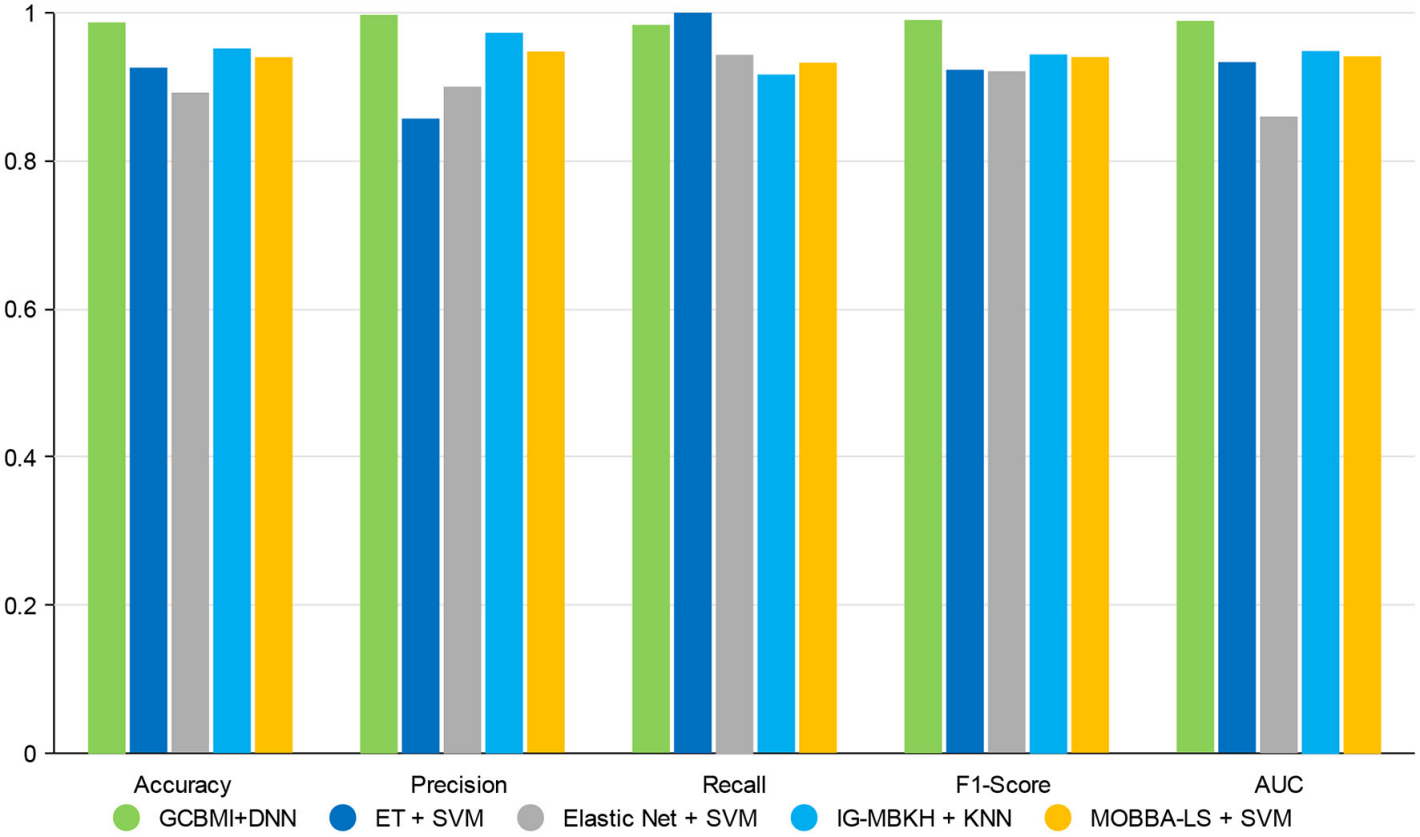
The experimental results of gastric cancer biomarker identification approach (GCBMI) compared with other methods.

F1-score and AUC value are often applied to evaluate the stability and robustness of models. The two indicators of GCBMI can achieve about 99%. It is 5–7% higher than other state-of-art methods. In order to display the advantages of our method more intuitively, the histogram of experimental results is plotted in Figure 3.

Overall, GCBMI can get better performance on different evaluation indicators than other feature selection methods, which indicates that the genes identified by GCBMI have more sufficient capacity to classify gastric cancer. The high F1-score and AUC value also illustrate that our model has better stability. The experimental results suggest that combined omics data are meaningful, and it may reveal some causal relationships between different biological layers.

### 5.2. The Impact of Classifiers on Performance

In this section, the impact of different classifiers is evaluated on our feature selection method. Table 4 displays the experimental results, which indicates that DNN model compared with the other three classifiers has better performance in different evaluation indicators. The performance of KNN is similar to SVM and NB is worst but still reaches 96%. The performance of our method is stable in different classifiers. GCBMI integrates gene expression data and DNA methylation data and expands the number of samples. In this way, the DNN model can be trained better and achieves superior results than other classifiers.

**Table 4:** Results with different classifiers (the accuracy, precision, recall, F1-score, and AUC value are average).

**Classifiers**	**Accuracy**	**Precision**	**Recall**	**F1-score**	**AUC**
DNN	**0.9870**	**0.9971**	**0.9836**	**0.9903**	**0.9891**
KNN	0.9776	0.9934	0.9729	0.9830	0.9795
SVM	0.9819	0.9878	0.9826	0.9862	0.9803
NB	0.9651	0.9698	0.9777	0.9737	0.9557

*The bold values represent the highest value of each metrics*.

**Figure 4:**
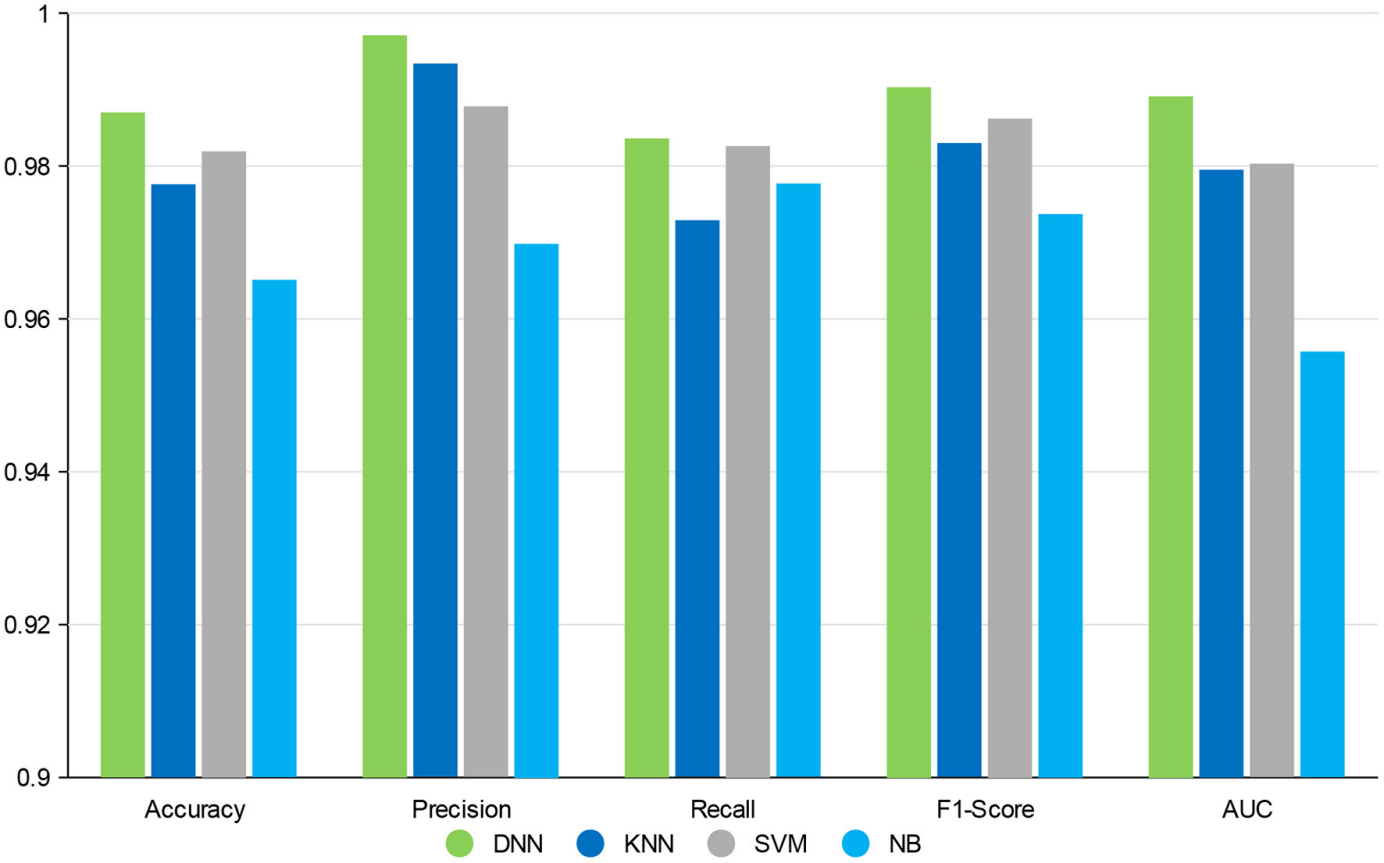
The experimental results of gastric cancer biomarker identification approach (GCBMI) with different classifiers.

On the whole, when compared with the KNN, SVM, and NB, our deep neural network model has better performance in different metrics, which indicates the validity of our feature selection approach. All the experimental results indicate that DNN model is a more appropriate classifier to feature selection in our approach. Figure 4 shows the histogram of the average accuracy, F1 score, and AUC value of GCBMI with different classifiers, respectively. The classification advantage of DNN model has been shown in it, which has demonstrated the effectiveness of GCBMI.

### 5.3. Gene Analysis

In our experiment, the overlapped genes are recorded, which are shown in Table 5. In each fold of cross-validation, about 20 genes are selected. These genes are the intersections of DEG and DMP. Among them, eight genes appear in each intersection and they are thought to be biomarkers of gastric cancer. In this section, the selected genes are further analyzed to understand the biological relevance.

**Table 5:** Selected genes from integrating gene expression and DNA methylation dataset.

**K-fold**	**Number of overlapping genes**	**Selected genes**
*K* = 1	17	FAHD2A,PGC,FIGF,PPAP2B,FOXA1,IFITM2,HOXC10, GPRC5C,CLEC3B,FBN1,LIF,C5,PSCA,PDGFD,KCNE2, RORC,C3
*K* = 2	19	PGC,FIGF,NID2,PPAP2B,IFITM2,RAB31,RORC,GPRC5C, FSCN1,TEAD4,CLEC3B,RAB17,IGFALS,C5,PSCA,PDGFD, KCNE2,COL4A1,C3
*K* = 3	17	FAHD2A,PGC,PPAP2B,FOXA1,IFITM2,IGFALS,GPRC5C, TEAD4,DNM1,ORM1,PTPRN2,FBN1,PSCA,PDGFD, KCNE2,RORC,C3
*K* = 4	24	PGC,FIGF,PDGFRB,PSMA7,TEAD4,C5,RORC,ADA, IFITM1,FAHD2A,PPAP2B,IGFALS,SLC1A2,GPRC5C, CLEC3B,CAPN9,KCNE2,PSCA,IFITM2,FSCN1,RPRM, PDGFD,SERPINA4,FBN1
*K* = 5	17	IFITM1,PGC,FIGF,PPAP2B,KCNE2,IFITM2,HOXC10, GPRC5C,CAPN9,FBN1,HRAS,C5,PSCA,PDGFD, SERPINA4,RORC,C3
Overlapped genes in 5-CV	8	PGC,RORC,GPRC5C,PDGFD,KCNE2,PSCA,IFITM2, PPAP2B

Through literature retrieving, we can find the coding protein of PGC is a digestive enzyme produced by the stomach and it is the main component of the gastric mucosa. Polymorphism of this gene is associated with gastric cancer susceptibility. Serum levels of this enzyme are used as the biomarker for certain stomach diseases, including *Helicobacter pylori* associated gastritis (Sun et al., 2009). Moreover, Liu et al. discovered PGC was positively expressed in normal gastric mucosa (100%), and the expression rate was 6.45% in gastric cancer (Liu D. et al., 2009). The results suggested that PGC has important application value in the diagnosis of gastric cancer.

For gene PSCA, relevant research demonstrated that proteins encoded by PSCA play an important role in cell proliferation. In addition to being highly expressed in the prostate, it is also expressed in differentiating gastric epithelial cells. This gene includes a polymorphism that results in an upstream start codon in some individuals; this polymorphism is thought to be associated with a risk for gastric cancers (Bahrenberg et al., 2000; Sakamoto et al., 2008).

Except for PGC and PSCA, gene PDGFD as a member of PDGF family (Huang et al., 2014), its signaling pathway has been considered as a new target for the treatment of gastric cancer (Wang et al., 2009). Besides, gene KCNE2 is expressed mainly in the cytoplasm of parietal cells. Kuwahara et al. discovered that the loss of KCNE2 expression could cause gastric adenocancer (Kuwahara et al., 2013).

**Figure 5:**
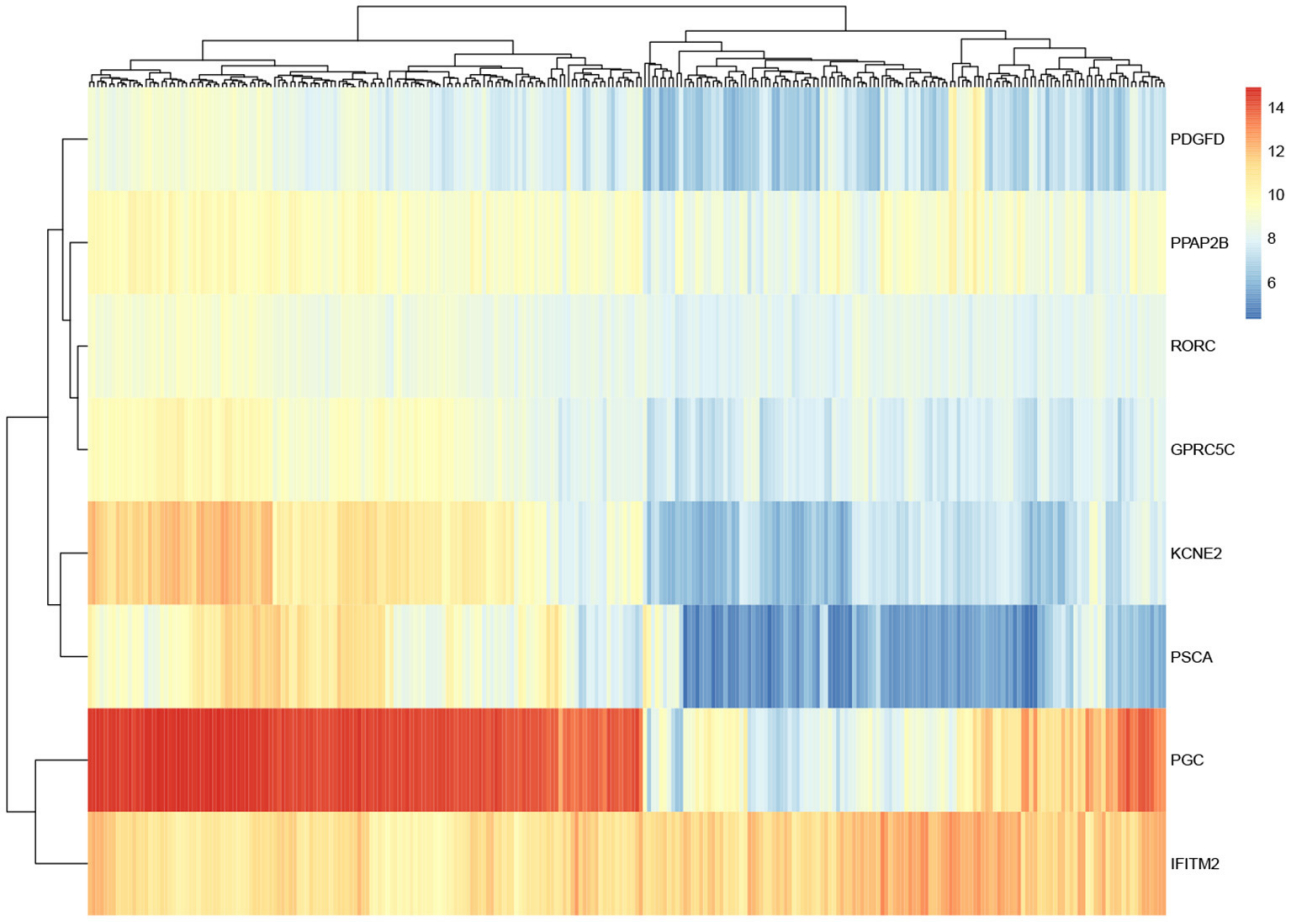
Heatmap of eight overlapped genes.

For these eight genes identified, in order to observe their expression level, gene expression heatmap is constructed. As shown in Figure 5, the expression levels of these eight genes in all samples are demonstrated. The first half of the heatmap are normal samples, and others are tumor samples. Basically, the result shows that these genes have different expression in normal and tumor samples. Some of these genes differed significantly between the two classes and may have some relationship with gastric cancer.

What is more, the enrichment analysis is conducted by DAVID database for selected genes. As shown in Table 6, biological significance of the genes are reported through Gene Ontology (GO). “GO:0008284 positive regulation of cell proliferation,” “GO:0046597 negative regulation of viral entry into host cell,” “GO:0030335 positive regulation of cell migration” are common biological activities in human cancer (Dyrskjøt et al., 2009). Among them, there have some items about platelet, some studies have suggested that gastric cancer may lead to changes in platelet count and morphology (Matowicka-Karna et al., 2013). In addition, some studies also have been pointed out that interferon (Ferrantini et al., 2007) and other related factors may have relationship with the occurrence of cancer.

**Table 6:** GO analysis of selected genes.

**Category**	**Term**	***p*-value**	**Gene**
GOTERM_BP_DIRECT	GO:0071560 cellular response to transforming growth factor beta stimulus	0.003912643	CLEC3B,FBN1, PDGFD
GOTERM_BP_DIRECT	GO:0043406 positive regulation of MAP kinase activity	0.005625548	HRAS,PDGFRB, PDGFD
GOTERM_BP_DIRECT	GO:0008284 positive regulation of cell proliferation	0.01138237	LIF,HOXC10,HRAS, PDGFRB,PDGFD
GOTERM_BP_DIRECT	GO:0002576 platelet degranulation	0.016395992	ORM1,CLEC3B, SERPINA4
GOTERM_BP_DIRECT	GO:0035456 response to interferon-beta	0.017024892	IFITM1,IFITM2
GOTERM_BP_DIRECT	GO:0035455 response to interferon-alpha	0.018899122	IFITM1,IFITM2
GOTERM_MF_DIRECT	GO:0048407 platelet-derived growth factor binding	0.020021643	COL4A1,PDGFRB
GOTERM_MF_DIRECT	GO:0005102 receptor binding	0.026443684	LIF,C3,C5,PDGFRB
GOTERM_MF_DIRECT	GO:0005161 platelet-derived growth factor receptor binding	0.02720561	PDGFRB,PDGFD
GOTERM_BP_DIRECT	GO:0036120 cellular response to platelet-derived growth factor stimulus	0.033768846	PDGFRB,PDGFD
GOTERM_BP_DIRECT	GO:0046597 negative regulation of viral entry into host cell	0.033768846	IFITM1,IFITM2
GOTERM_BP_DIRECT	GO:0030335 positive regulation of cell migration	0.047784333	HRAS,PDGFRB, PDGFD
GOTERM_BP_DIRECT	GO:0048008 platelet-derived growth factor receptor signaling pathway	0.053858697	PDGFRB, PDGFD

## 6. Conclusion

In this work, we propose a novel feature selection approach, GCBMI, which uses gene expression and DNA methylation data for identifying the biomarkers of gastric cancer. GCBMI consists of three main parts, namely data pre-processing, selection of differentially expressed genes and data combination, and deep neural network as the classifier. Differential expression analysis, statistical test, and MI are integrated to obtain comprehensive view to implement the biomarkers identification after data pre-processing. MI is introduced to filter out irrelevant gene, and FC and *T*-test are utilized to select differentially expressed genes. In particular, FDR is applied to revise the *p*_value to further screen genes. After that, Cartesian product is performed to expand samples. Moreover, GCBMI adopts DNN as the classifier to evaluate the classification ability of selected genes. Experimental results on GEO dataset indicate that the proposed approach outperforms other state-of-the-art feature methods. The results of biological relevant verification indicate the status of the selected gene as the biomarkers of gastric cancer.

What is more, the performance of combined with omics data tends to be more superior than using a single omics data alone. In the future, some other omics data will be combined such as copy number variation (CNV) data to identify cancer biomarkers, and our methods will be applied to other fields as well (Liu et al., 2020). Besides, some measures will also be taken to improve our method so that its classification performance can be improved further.

## Data Availability Statement

The original contributions presented in the study are included in the article/supplementary material, further inquiries can be directed to the corresponding author/s.

## Author Contributions

CY and ZX conceived and designed the approach. ZX performed the experiments. HL analyzed the data. GZ and ZX wrote the manuscript. CY and JW supervised the whole study process and revised the manuscript. All authors have read and approved the final version of manuscript.

## Conflict of Interest

The authors declare that the research was conducted in the absence of any commercial or financial relationships that could be construed as a potential conflict of interest.
